# Citywide serosurveillance of the initial SARS-CoV-2 outbreak in San Francisco using electronic health records

**DOI:** 10.1038/s41467-021-23651-6

**Published:** 2021-06-11

**Authors:** Isobel Routledge, Adrienne Epstein, Saki Takahashi, Owen Janson, Jill Hakim, Elias Duarte, Keirstinne Turcios, Joanna Vinden, Kirk Sujishi, Jesus Rangel, Marcelina Coh, Lee Besana, Wai-Kit Ho, Ching-Ying Oon, Chui Mei Ong, Cassandra Yun, Kara Lynch, Alan H. B. Wu, Wesley Wu, William Karlon, Edward Thornborrow, Michael J. Peluso, Timothy J. Henrich, John E. Pak, Jessica Briggs, Bryan Greenhouse, Isabel Rodriguez-Barraquer

**Affiliations:** 1 University of California San Francisco, San Francisco, CA USA; 2 Chan Zuckerberg Biohub, San Francisco, CA USA

**Keywords:** Computational models, SARS-CoV-2, Viral infection, Epidemiology

## Abstract

Serosurveillance provides a unique opportunity to quantify the proportion of the population that has been exposed to pathogens. Here, we developed and piloted Serosurveillance for Continuous, ActionabLe Epidemiologic Intelligence of Transmission (SCALE-IT), a platform through which we systematically tested remnant samples from routine blood draws in two major hospital networks in San Francisco for SARS-CoV-2 antibodies during the early months of the pandemic. Importantly, SCALE-IT allows for algorithmic sample selection and rich data on covariates by leveraging electronic health record data. We estimated overall seroprevalence at 4.2%, corresponding to a case ascertainment rate of only 4.9%, and identified important heterogeneities by neighborhood, homelessness status, and race/ethnicity. Neighborhood seroprevalence estimates from SCALE-IT were comparable to local community-based surveys, while providing results encompassing the entire city that have been previously unavailable. Leveraging this hybrid serosurveillance approach has strong potential for application beyond this local context and for diseases other than SARS-CoV-2.

## Abstract (web summary)

Population-based surveys are the gold standard for estimating seroprevalence but are expensive and often only capture a small geographic area or window of time. This study describes a new platform, SCALE-IT, for serosurveillance based on algorithmic sampling of electronic health records, and uses it to estimate the seroprevalence of SARS-CoV-2 in San Francisco.

## Introduction

The rapid spread of the SARS-CoV-2 virus has laid bare important gaps in routine infectious disease surveillance. Serological data, particularly when collected at high spatial and temporal resolutions, are a key resource for addressing many key epidemiological questions since they directly quantify the proportion of the population that has been infected by a pathogen^1,2^. For SARS-CoV-2, serology is particularly useful given the high levels of disease under-ascertainment: serologic surveillance is the gold standard for estimating attack rates (the proportion of the population that has been infected) and highly complementary to virologic and syndromic surveillance systems for providing vital information on where a population is along the epidemic curve^3^. Population-based serosurveys that employ a probabilistic sampling frame are considered to be the gold standard for estimating seroprevalence. However, performing large population-based serosurveys can be prohibitively resource-intensive to initiate swiftly or perform repeatedly, especially during an ongoing outbreak, as demonstrated by the relative sparsity of population-based versus convenience sampled serosurveys for SARS-CoV-2 that have been conducted to date^3^. For example, to date, no population-based serosurveys have been conducted for the city of San Francisco or wider Bay Area, and few have been conducted in the United States, limiting our ability to identify of risk factors for infection, understand population-level immunity, and determine which populations and localities may be in need of targeted public health resources such as testing, contact tracing, or vaccine allocation^4^.

Residual blood samples from readily available sources (e.g., blood donors or remnant samples collected from routine medical care visits), especially when linked to individual-level meta-data, provide a unique opportunity to address these limitations and to efficiently survey a population for antibodies over an extended period of time^5,6^. Such studies were found to be useful in the 2009 H1N1 influenza pandemic^7–13^, facilitating analyses on a broader spatial and temporal scale than typical cross-sectional serological surveys allow. However, in most studies that use residual blood samples the source population is unknown^14^. This presents a major limitation, as the results are difficult to interpret when it is not known whether the sampled population is representative of the population of interest.

The San Francisco Bay Area has widely been recognized for taking an early and proactive response to COVID-19. San Francisco Bay Area counties introduced a shelter-in-place order on 17 March 2020, requiring residents to remain at home unless leaving the house for essential activities. Relative to many other US cities, few cases were detected in San Francisco during the early months of the epidemic, a pattern which continued as the pandemic progressed^15^. However, like many other areas, a high proportion of asymptomatic infections and limited access to diagnostic testing during this time makes it difficult to interpret these numbers. Results from an early San Francisco seroprevalence study conducted on convenience samples in late March to early April 2020 suggested that <1% of the population had been infected overall^16^, in contrast to a seroprevalence of >6% estimated by a community study focusing on a specific neighborhood, particularly among the Hispanic/Latinx population^17^ but consistent with a survey of a rural Bay Area community^18^. The lack of citywide, representative seroprevalence estimates during this time period limits the ability to determine to what degree these discrepancies reflect heterogenous exposure or differences in study design.

Here we present a blueprint and the early results of the ongoing SCALE-IT study (*Serosurveillance for Continuous, ActionabLe Epidemiologic Intelligence of Transmission*), leveraging residual sera samples from two large hospital systems in San Francisco, California to quantify the prevalence of SARS-CoV-2 antibodies. Importantly, these remnant samples are linked to electronic health records (EHRs) enabling careful algorithmic selection based on demographic and clinical variables, improving their representativeness to the general population. We tested over 5000 samples collected from late March to June 2020 from San Francisco residents, and calculated raw and adjusted seroprevalence estimates over space, time, and socio-demographic indicators. These data provide estimates of the overall seroprevalence in San Francisco during the initial phase of the local SARS-CoV-2 outbreak and highlight spatial and demographic heterogeneities in transmission across the city.

## Results

**Fig. 1:**
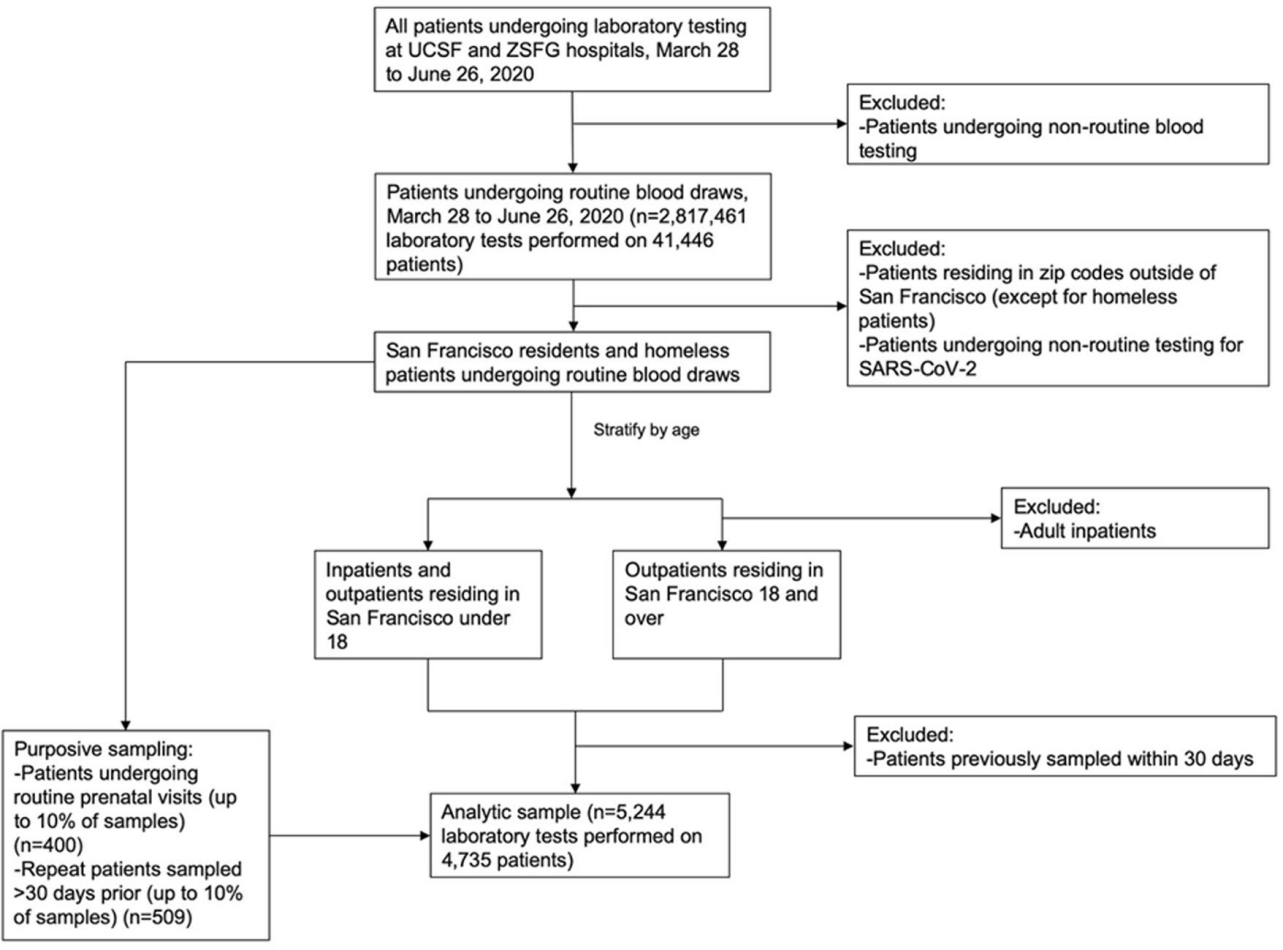
Flow diagram of sampling algorithm. Flow diagram showing the inclusion and exclusion criteria used at different stages of the data screening and sample selection process.

**Fig. 2:**
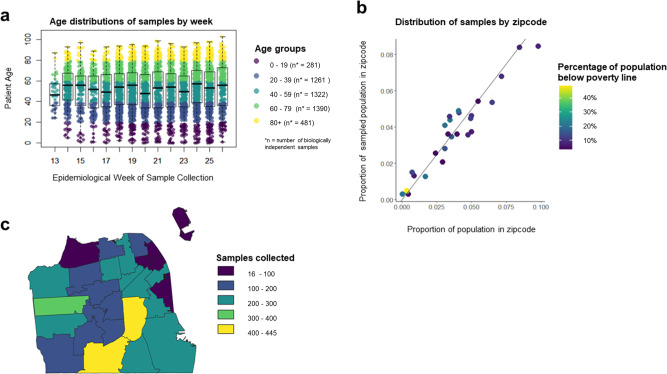
Distributions of SCALE-IT samples. Figure showing distributions of SCALE-IT samples **a** boxplot showing the distribution of samples by epidemiological week and age group (whiskers show minimum and maximum age, box shows 0.25 quantile, median and 0.75 quantile age respectively) **b** plot of the proportion of the samples collected from patients residing in a zip code, plotted against the actual proportion of the San Francisco population living in that zip code. Colors show the proportion of the population in that zip code living below the poverty line using the 2018 American Community Survey thresholds, and **c** map of counts of samples collected by zip code.

Between March 28, 2020, and June 26, 2020, we collected a total of 5244 samples, representing 4735 individual patients, from UCSF Health (*n* = 3037 patients) and ZSFG (*n* = 1698 patients) (Fig. 1, Supplementary Fig. 1). By design, the age distribution of sampled individuals remained consistent throughout the study period, and the geographic distribution of residents matched the proportion of the San Francisco population living in each zip code (Fig. 2). Our sample did not achieve the target sample size for the youngest age group due to the limited number of children receiving routine phlebotomy in the UCSF and ZSFG health systems (Table 1). Our results were relatively representative of the San Francisco population by race and ethnicity, although our sample overrepresented those who identified as Black/African American and slightly underrepresented those who identified as Asian.

**Table 1:** Distribution of Socio-demographic characteristics of patients sampled. Table showing socio-demographic characteristics of patients sampled in SCALE IT and of the San Francisco population (2019).

	UCSF (*n* = 3037)	ZSFG (*n* = 1698)	Total sampled individuals (*n* = 4735)	SF Population (ACS 2019)
Sex				
Female	1733 (57.1%)	758 (44.6%)	2491 (52.6%)	49.3%
Male	1302 (42.9%)	929 (54.7%)	2231 (47.1%)	50.8%
Unknown	2 (0.1%)	11 (0.6%)	13 (0.3%)	N/A
Age				
0–19	246 (8.1%)	35 (2.1%)	281 (5.9%)	15.0%
20–39	836 (27.5%)	425 (25.0%)	1261 (26.6%)	38.0%
40–59	731 (24.1%)	591 (34.8%)	1322 (27.9%)	25.3%
60–79	834 (27.5%)	556 (32.7%)	1390 (29.4%)	17.3%
80+	390 (12.8%)	91 (5.4%)	481 (10.2%)	4.3%
Race/Ethnicity				
American Indian or Alaska Native	3 (0.1%)	9 (0.5%)	12 (0.3%)	0.3%
Asian	783 (25.8%)	423 (24.9%)	1,206 (25.5%)	34.6%
Black or African American	283 (9.3%)	308 (18.1%)	591 (12.5%)	5.2%
Other	214 (7.0%)	73 (4.3%)	287 (6.1%)	4.5%
Other Pacific Islander	28 (0.9%)	17 (1.0%)	45 (1.0%)	0.4%
White	1317 (43.4%)	358 (21.1%)	1675 (35.4%)	39.8%
Unknown or declined	43 (1.4%)	18 (1.1%)	61 (1.3%)	N/A
Hispanic^a^	366 (12.1%)	492 (29.0%)	858 (18.1%)	15.2%
Insurance type				
Uninsured	119 (3.9%)	150 (8.8%)	269 (5.7%)	N/A
Government	1462 (48.1%)	1475 (86.9%)	2937 (62.0%)	N/A
Private or employer	1351 (44.5%)	70 (4.1%)	1421 (30.0%)	N/A
Unknown	105 (3.5%)	3 (0.2%)	108 (2.3%)	N/A

^a^Hispanic includes respondents of any race. Other categories are non-Hispanic.

Overall, from 5244 samples we identified 192/4735 positive samples from unique patients for a raw seroprevalence of 4.1%. After weighting for age group and sex to match the population structure of San Francisco and correcting for test performance characteristics (overall sensitivity of 93.7% and specificity of 99.6%), this corresponds to an estimated population seroprevalence of 4.2% (95% Credible Interval [CrI]: 2.1–6.3%). Based on the number of cases reported during the period covered by the study, we estimate that only 4.9% of all infections were ascertained by the reporting system (95% CrI: 3.3–9.9%) (Supplementary Methods 1). Amongst pregnant women seeking routine care (*N* = 268), we estimated a raw seroprevalence of 3.4% (9/268 seropositive), and after adjusting for test performance characteristics we estimate 3.5% (95% CrI: 1.1–6.4%) seroprevalence amongst this group. This estimate in our sentinel population group is consistent with the estimates across our overall population of samples.

**Fig. 3:**
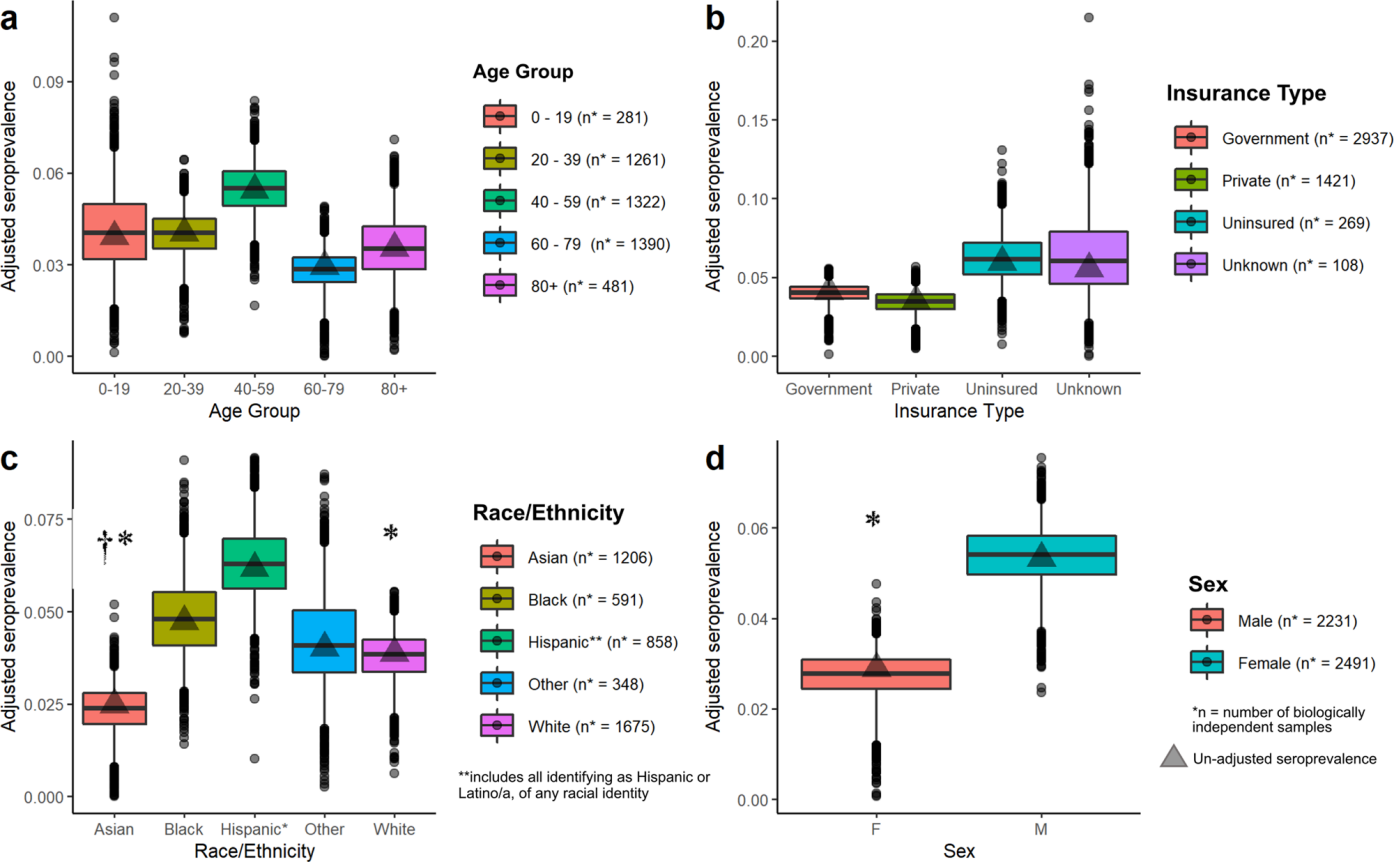
Stratified seroprevalence by demographic group. Box and whisker plots showing posterior estimates of seroprevalence from *n* = 7500 iterations of the algorithm to produce adjusted estimates for test performance (Supplementary Methods1), stratified by **a** age, **b** insurance type, **c** race/ethnicity (groups containing *n* < 50 samples were included in ‘other’) and **d** sex. The shaded triangle shows the raw seroprevalence estimate. The number of biologically independent samples used to calculate raw and adjusted seroprevalence estimates for each stratified group are shown in the figure legends. The midline of the boxplot shows the median of the posterior, the upper and lower edges of the box show the 25% and 75% quantiles, whiskers show 95% credible interval of the posterior. Points show posterior estimates outside of this interval. For (**c**), stars (*) indicate the race/ethnic groups where the 2.5% and 97.5% quantiles of the differences in posterior estimates for seroprevalence between samples from Hispanic patients and that group did not cross zero. Crosses (†) indicate the ethnic groups where the 2.5% and 97.5% quantiles of the differences in posterior estimates for seroprevalence between samples from Black or African American patients and that group did not cross zero. For (**d**) a star (*) indicates that the 2.5% and 97.5% quantiles of the differences in posterior estimates for seroprevalence between Males and Females did not cross zero.

We did not observe statistically significant differences in seroprevalence by age (Fig. 3a) or hospital system (Supplementary Table 2, Supplementary Data 1). We found seroprevalence to be nearly twice as high in uninsured individuals (6.3%, 95% CrI: 3.1–9.9%) than in those with some form of insurance, [Private/Commercial: 3.4% (95% CrI: 1.6–4.7%); Government: 4.0% (95% CrI: 2.3–5.0%)] (Fig. 3b). With respect to race/ethnicity, seroprevalence was highest in those identifying as Hispanic (6.3%, 95% CrI: 4.4–8.3%) followed by Black or African American (4.8%, 95% CrI: 2.8–7.0%), and lowest in those who identified as Asian (2.3%, 95% CrI: 0.8–3.5%) (Fig. 3c). Seroprevalence was almost twice as high in those identifying as Male (5.3%, 95% CrI: 3.7–6.6%) compared to Female (2.7%, 95% CrI: 1.1–3.6%) (Fig. 3d). Although these samples were obtained over a 3-month collection period, given the relatively low attack rate during these initial stages of the pandemic in San Francisco, we were not able to detect meaningful differences in seroprevalence over time (Supplementary Table 2, Supplementary Figs. 2 and 3).

**Fig. 4:**
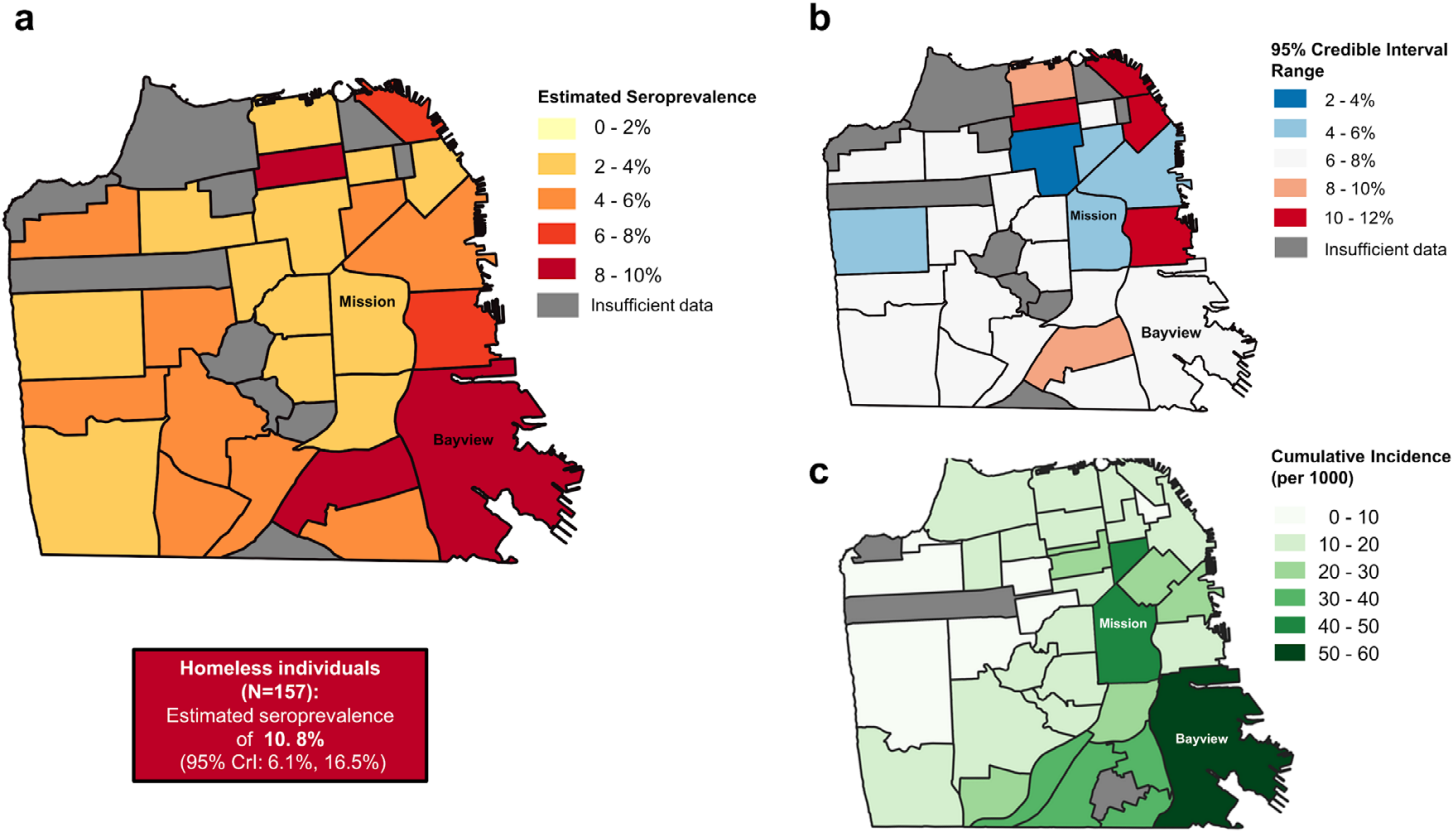
Multi-panel map of seroprevalence by geography. Maps show **a** seroprevalence by neighborhood, adjusted for test performance. Box shows adjusted seroprevalence in individuals experiencing homelessness. **b** range of 95% Credible interval of estimates, **c** cumulative incidence by planning neighborhood from March to June 2020, using data from San Francisco Department of Public Health (https://data.sfgov.org/COVID-19/COVID-19-Cases-by-Geography-and-Date/d2ef-idww). Estimates for neighborhoods with under 50 samples from unique individuals are not plotted and shown in gray.

Geographically, we found seroprevalence to be highest in the Bayview neighborhood in the southeast region of the city, at 8.1% (95% CrI: 4.6%, 12.3%) (Fig. 4a, Supplementary Table 3, Supplementary Data 2). Although several other neighborhoods had similarly high seroprevalences, there was much more uncertainty around these estimates (Fig. 4b). These findings are consistent with patterns of incidence in the city during this period of time (Fig. 4c). We identified 157 individuals who were homeless in our study, and amongst this group seroprevalence was estimated to be 10.8% (95% CrI: 6.1%, 16.5%).

As validation of the representativity of our approach using curated remnant samples, we compared results from this study to two contemporaneous community-based serosurveys conducted in specific neighborhoods of San Francisco. First, we compared these results to a cross-sectional serosurvey carried out in a census tract within the Mission District (census tract 022901, zip code 94110) between April 25 and April 28, 2020^17^. Chamie et al. tested 2545 census tract residents for SARS-CoV-2 antibodies and estimated seroprevalence to be 3.1% (95% CI: 2.5–3.9%). This is consistent with our findings of 3.8% seroprevalence (95% CrI: 1.8–6.3%) between April and June 2020 in the broader Mission District neighborhood. Second, we compared our results to a cross-sectional serosurvey carried out in two census tracts in San Francisco’s 10th District between May 30 and June 2, 2020 (https://unitedinhealth.org/sf-district-10), located in the Bayview neighborhood. Among the nearly 1600 individuals tested for antibodies, seroprevalence was estimated at 5.6% in Hispanic participants (*n* = 320), 2.3% in Black participants (*N* = 397) and 0.4% in white participants (*n* = 231). The relatively high seroprevalence we detected in the Bayview neighborhood through our study is comparable to the results of this community-based study, and the disparities by race/ethnicity were similar in direction, though different in magnitude, to those identified through our remnant sample study as well. It is worth noting that the community studies available for comparison also rely upon convenience sampling as participation in the studies was voluntary, and therefore may contain inherent selection biases themselves.

## Discussion

In this study, we developed and piloted a scalable and systematic pipeline using remnant samples from two major hospital networks in San Francisco to select, collect, and test specimens for SARS-CoV-2 antibodies (SCALE-IT). Through this effort, we estimated seroprevalence during the early months of the epidemic to be relatively low throughout San Francisco (4.2%), but still representing more than 20 times the number of infections identified by PCR-confirmed cases at that time. This may be due to the limited availability of PCR testing during the beginning of the pandemic, and the lack of testing of asymptomatic individuals. We also identified important disparities in seroprevalence at the neighborhood level, with the highest seroprevalence in the Bayview neighborhood in the southeast region of the city, as well as disproportionately higher seroprevalence in individuals experiencing homelessness and those identifying as Hispanic, Black/African American, or male. Leveraging this hybrid serosurveillance approach has potential for broad application beyond this local context and for diseases other than SARS-CoV-2.

The heterogeneities in seroprevalence we observed by race/ethnicity and socio-economic status—here obtained from EHR data on health insurance status and whether individuals were housed—echo the patterns, which have been highlighted over the course of the pandemic at national and global levels^19,20^. Specific to San Francisco, our results provide estimates of SARS-CoV-2 cumulative exposure at a granular spatial resolution with a scope covering the entire city; despite low overall seroprevalence, we identified specific neighborhoods with disproportionately higher seroprevalence. Interestingly, we also found seroprevalence to be approximately twice as high in those identifying as male compared to female. Potential explanations for this difference include differential pathogen exposure by sex, which is supported by findings of other studies elsewhere^14^ and in San Francisco, finding PCR positivity rates of 1.2% (20/1658) in women and 3.3% (63/1908) in men, with an odds ratio of 2.71 (1.64-4.69) for PCR positivity in males, and also that the majority (74%,) of those who tested positive by PCR or were seropositive for SARS-CoV-2 were frontline workers and unable to shelter-in-place^17^. It has been found that males and females mount different immune responses and infection severity^21^, which could affect assay sensitivity, however, we believe this is unlikely to explain the large difference we see in our estimates as we do not see sex-based differences in the sensitivity of our assay on the positive controls used in the study, which represent a range of disease severities.

While a key strength of our approach was leveraging residual sera from two large health system networks and using data from EHRs to algorithmically select samples for inclusion, there are limitations to this type of surveillance that require consideration. Most obviously, patient samples may not be fully representative of the underlying population. This may be particularly true during “shelter-in-place” periods, when behavioral changes may affect the availability and characteristics of the patient population. These issues can ideally be mitigated by careful sample selection, as done here by focusing on a subset of outpatients, with the possibility of further refinement by inclusion of additional selection criteria (e.g., by restricting or weighting sampling to consider specific visit types or underlying conditions). Representativity of the serosurveillance system could also be enhanced by including a broader network of local health systems. We also recognize that the generalizability of our findings may differ by age groups, and is likely to be lower in children who were underrepresented in our sample set despite the stratified sampling framework. Additional study designs, such as school-based serosurveys, could be leveraged to augment these data to prospectively assess seroprevalence in specific age groups, possibly by using non-invasive, saliva-based antibody testing^22^. Despite including over 5000 samples, our study was not powered to detect differences between covariates or by time in a multiple regression framework, in part due to San Francisco’s success in maintaining low transmission and thus low seroprevalence during this time period. Lastly, while we validated our estimates against results from available community-based studies, further validation would be ideal to assess validity of results and findings.

Whilst our estimates of seroprevalence in the Mission and Bayview districts were consistent with community studies and we found similar disparities by demographics, we did find slightly higher seroprevalences overall. This was particularly true for the Bayview/Sunnydale surveys where we estimate a seroprevalence of 8.1% (95% CrI: 4.6–12.3%) for Bayview/Hunter’s point neighborhood, whilst a community survey in the census tract 231.02 which lies within the neighborhood, found a raw seroprevalence estimate of 24/784 (3.06%). This difference may be due to heterogeneity within the neighborhood, i.e., higher seroprevalences in other census tracts not sampled in the community survey, or differences in the underlying population sampled. There are also differences in the timing of sample collection; we collected samples up until the end of June 2020, whereas this study was conducted between May 30 and June 2, 2020. In addition, the difference could be caused by our study sampling individuals more at risk of exposure than the community surveys. It is also interesting to compare our results to other serosurveys, which sampled the wider San Francisco Bay Area during the early months of the pandemic. A serosurvey across the wider San Francisco Bay Area found a seroreactivity 0.1% in 1000 blood donors, and 0.26% in 387 hospitalized patients admitted for non-respiratory indications in early April 2020^23^. An additional study of residual sera in the San Francisco Bay area between 23 and 27 April found a seroprevalence of 1.0%^14^. These results are quite a bit lower than our estimate for April of 4.6% (2.7–6.3%), but not directly comparable as the source populations drawn for these studies are not fully characterized and are unlikely to be representative of the San Francisco population. In addition, samples in both studies included residents from outside of San Francisco county, including counties known to have experienced very low transmission of SARS-CoV-2 during this time period.

We did not find a clear increase in seropositivity over time, whilst case counts in San Francisco did increase, albeit slowly, during the observation period. This lack of increase in seroprevalence over time may be the result of changes in some of the demographics of our sample population over time (Supplementary Fig. 2, Supplementary Table 4), as the proportion of samples from patients who identify as white, female and who have private insurance (all of which we found had lower seroprevalence) increased over the period of sample collection. This could also be explained by a lack of power to detect small changes at such low seroprevalence. If implemented in a context where there was more power to detect changes and/or stratify by additional demographic variables when selecting samples, then our approach could provide valuable data to explore additional questions of public health interest, such as the impact of interventions and changes in ascertainment rates over time.

In this pilot study, we developed and implemented a SARS-CoV-2 serosurveillance system to detect population-level pathogen exposure in near-real time, and demonstrated how data collected through this platform were comparable to results from more resource-intensive community-based serological studies and incidence data. The appeal of this hybrid approach is that it achieves many of the strengths of population-based surveys and provides rich data, while leveraging existing infrastructure to allow for much greater efficiencies often seen in convenience sampling approaches. Using EHR data, we were able to develop a stratified sampling frame, ensuring improved representativeness of the results in contrast to serosurveys performed using convenience samples without these key pieces of information^14^. At the same time, we used these data to identify important spatial and demographic heterogeneities in seroprevalence within our study site; serosurveys performed on residual samples are often limited to coarser levels of meta-data on the sampled population^24^. The relative ease with which SCALE-IT can be implemented means that it can be deployed over a broad geographic scale, continuously over time, and dynamically adjusted to address specific surveillance needs.

We envision multiple lines of work for future directions. First, the samples that we have selected, collected, and processed in this work could serve as a valuable biorepository for future applications. The ability to link rich EHR data to a large bank of well-curated serum samples opens up opportunities for additional analysis including longitudinal studies of patients. Second, as serosurveillance efforts will be fundamental to monitor SARS-CoV-2 transmission rates and evaluate the impact of control interventions (both Non Pharmaceutical Interventions and pharmaceuticals) over the coming months and years, future work could leverage these and prospective serological data to parametrize mechanistic models and to study the effects of control strategies on infection rate. Third, as discussed by others^1,2^, our local SCALE-IT platform could easily be expanded to contribute to a ‘Global Immunological Observatory’ to perform serosurveillance for other pathogens beyond the SARS-CoV-2 virus. Data generated by such an observatory could be used to address specific public health gaps including serosurveillance for seasonal pathogens such as influenza or emerging infections. Lastly, the insights gained from developing this platform could serve as a blueprint for adoption by other health systems in various contexts.

## Methods

### Data source

Residual serum samples from routine blood draws from the University of California, San Francisco (UCSF) and San Francisco Department of Public Health (SFDPH) inpatient and outpatient healthcare systems were sampled from March 28, 2020, onward. UCSF Medical Center is a network of three hospitals with ~1.8 million outpatient visits annually (https://www.ucsfhealth.org/about/annual-reports). The SFDPH hospital, Zuckerberg San Francisco General Hospital (ZSFG), is a city hospital that provides trauma, medical, and surgical services to a heterogeneous population of largely un- or underinsured patients, including the city’s homeless population, and serves roughly 100,000 patients per year (https://zsfg.ucsf.edu/about-ucsf-zsfg).

We obtained daily EHRs for all patients in these networks undergoing routine blood testing, defined as blood chemistries and tests for sexually transmitted infections and rubella. EHR data included information on patient demographics, address, insurance provider, and diagnoses. We also obtained information on all tests for respiratory infections (including SARS-CoV-2) performed on patients in the 6 months prior to the blood draw.

### Sampling methodology

We aimed to collect 2000 samples monthly. We determined this sample size based on considerations of both statistical power and feasibility. To estimate seroprevalence with an absolute error of 5% and at Type I error of 5%, and a prior of 20% seroprevalence, a sample size of 246 individuals would need to be tested each month. We determined that an overall sample size of a minimum 1230 samples per month would be sufficient to allow stratification of results by five age groups (0–19, 20–39, 40–59, 60–79, 80+ years).

From the full list of residual serum samples that were available, we restricted our sampling frame to samples from individuals undergoing routine blood testing. We included patients residing in San Francisco, including those experiencing homelessness. We excluded individuals who were tested for SARS-CoV-2 during the visit when they received their blood draw (except if the test was for routine purposes, such as testing prior to an elective procedure or admittance to the hospital). We did not have any exclusion criteria for previous visits or tests for SARS-CoV-2 of any severity. We restricted our sample to outpatient and emergency department visits for adults; for the youngest age group, we included both inpatient and outpatient visits due to small numbers of available samples. Finally, we excluded samples if a sample from the same patient had been selected within the previous 30 days.

After obtaining the list of eligible samples according to the above criteria, we selected serum samples for the study using a sampling algorithm aimed to ensure an adequate sample size for each of five age strata and to maximize geographic representativity. After setting a daily target sample size for our overall population, we divided this equally between five age bins to set a target sample size for each age bin. We also set a target sample size for each zip code, which was proportional to its population size. For each zip code with a larger number of eligible samples than its target size, we kept all samples from age groups with sample sizes below or at their target and obtained a random sample from any age group that had an eligible sample size above the target size. We intentionally oversampled pregnant women as a healthy sentinel population by aiming to obtain up to 10% of the samples from pregnant women undergoing routine care, as defined by ICD-10 codes.

### Sample processing

Remnant samples were stored at +4 °C in outpatient laboratories at UCSF and ZSFG, and collected by our study team twice every week. After collection, samples were centrifuged for 15 min at 3500 g before aliquoting a working stock of 300 μL into 96 well-barcoded tubes, diluting in 1:1 HEPES storage buffer, and storing at +4 °C. The remainder of the sample was aliquoted into 1.4 mL barcoded tubes and stored at −20 °C.

### Serologic assays and validation data

We used two serologic assays for this study in order to maximize assay specificity. First, we screened all samples using an in-house ELISA assay and then performed confirmatory testing on a subset of samples above a threshold value using an in-house Luminex assay. The ELISA assay detected IgG to the receptor-binding domain (RBD) of the spike (S) protein, based on published protocols^25^ with minor modifications, described here briefly. 1 μg of RBD was used to coat each well of 384-well high binding plates, secondary antibody was diluted 1:5000 (Southern Biotech #2048-05), and OPD was used to develop the plates. Concentration values were calculated from the ELISA optical density using a plate-specific standard curve from serial dilutions of a pool of positive control samples^26^. Samples with an ELISA concentration value above 0.049 were selected for confirmatory testing (see Supplementary Methods 1, Supplementary Tables 5 and 6).

For confirmatory testing, we used a multiplex microsphere assay (Luminex platform) to detect IgG against the SARS-CoV-2 S protein, RBD, and the nucleocapsid (N) protein, based on a standardized serology protocol with minor modifications^27^. Briefly, plasma samples were diluted to 1:100 in blocking buffer A (1× PBS, 0.05% Tween, 0.5% bovine serum albumin, 0.02% sodium azide). Antigen concentrations used were as follows: S: 4 μg/mL, RBD: 2 μg/mL, and N: 3 μg/mL. As above, concentration values were calculated from the Luminex median fluorescent intensity using a plate-specific standard curve from serial dilutions of a pool of positive control samples. A logistic regression model including the concentration values of the three antigens for each sample was determined to have the highest cross-validation accuracy for classification and was used to establish a cutoff for positivity (see Supplementary Methods 1).

Serologic assays were optimized using positive and negative controls from several sources. Serum samples from 127 patients with PCR-confirmed SARS-CoV-2 infections (representing 266 total samples, with 1-4 longitudinal monthly time points per individual beginning at 3 weeks post-symptom onset) were obtained from the *Long-term Impact of Infection with Novel Coronavirus* (LIINC) study (https://www.liincstudy.org/) and used as positive controls. Importantly, participants in this cohort represent a range of infection severities (ranging from asymptomatic to severe), age, sex, and ethnicity and race. Serum samples from 119 individuals obtained prior to the emergence of SARS-CoV-2 were used as negative controls. The overall sensitivity of our serial testing approach using positive and negative controls was 93.7% (95% CrI = 89.0%, 97.2%) and specificity was 99.6% (95% CrI = 98.2%, 100.0%) (Supplementary Tables 1, 5 and 6, Supplementary Methods 1).

### Analytic methods

Raw seropositivity was determined as the proportion of all samples from unique individuals that tested positive on the confirmatory assay. We then produced estimates of seroprevalence adjusted for the sensitivity and specificity of the serial testing approach, incorporating potential conditional dependence of the tests as described in Gardner et al.^28^ (see Supplementary Methods 1). We stratified by covariates to obtain seroprevalence estimates for each stratum (age, sex, insurance status, ethnicity, and neighborhood). To identify neighborhoods, we geocoded sample addresses using the Google Cloud Geocoding API using the *ggmap* R package^29^. Samples (*n* = 365 unique individuals) which could not be geocoded to rooftop (*n* = 261) and/or were from homeless individuals (*n* = 157) were excluded from neighborhood level estimates of seroprevalence, however, estimates of seroprevalence were calculated for homeless individuals separately and provided alongside neighborhood level estimates of seroprevalence. All analysis was conducted using the R statistical software^30^ and the Stan programming language^31^. Code and data to reproduce all analyses are available at: https://github.com/EPPIcenter/scale-it^32^.

### Institutional Review Board (IRB) approval

This study received expedited review approval by the UCSF IRB #20-30379 (Serological Surveillance of SARS-CoV-2 in Residual Serum/Plasma Samples). The IRB did not require patient contact or written consent to use residual sera. The LIINC study (providing positive control samples) was approved by the UCSF (IRB #20-30479). Pre-pandemic samples used as negative controls came from the New York Blood Bank, and were de-identified and not subject to IRB review for use in this study.

### Reporting summary

Further information on research design is available in the Nature Research Reporting Summary linked to this article.

## Supplementary information

Supplementary Information

Descriptions of Additional Supplementary Files

Dataset 1

Dataset 2

Reporting Summary

## Data Availability

To avoid identifiability of data and to comply with institutional policy around data privacy, we have provided summarized data by demographic group and neighborhood instead of individual-level data, used to generate Fig. 4a, b, as well as posterior values for seroprevalence by demographic group used to generate Fig. 3a–d. The aggregated data used for this analysis can be found on Github at https://github.com/EPPIcenter/scale-it/ (DOI:10.5281/zenodo.4695335)^26^. Maps were created in QGIS (QGIS.org, QGIS Geographic Information System. QGIS Association. http://www.qgis.org, 2021) using shapefiles in the public domain (Fig. 2c: California. Metropolitan Transportation Commission. Census Zip Code Tabulation Areas, 2000 - San Francisco Bay Area, California. Retrieved from https://earthworks.stanford.edu/catalog/stanford-df986nv4623, 2002) (Fig. 4a–d: City of San Francisco, SF data (2019) Planning Neighborhood Groups Map, https://data.sfgov.org/Geographic-Locations-and-Boundaries/Planning-Neighborhood-Groups-Map/iacs-ws63, 2019). Cumulative incidence by planning neighborhood from March - June 2020 in Fig. 4c used publicly available data from the San Francisco department of Public Health (https://data.sfgov.org/COVID-19/COVID-19-Cases-by-Geography-and-Date/d2ef-idww). Figures 3 and 4 visualize Supplementary Tables 2 and 3. Figure 2 visualizes the distribution of samples, although because the underlying raw data for Fig. 2 are at the individual level, they have not been shared with the manuscript for ethical reasons, although the summarized demographic distributions of the samples are included in the manuscript (Table 1) and access to full raw data can be requested from the authors by contacting Bryan Greenhouse. Data for poverty rates shown in Fig. 2c come from the American Community Survey 2019 (https://data.census.gov/cedsci/).

## Code availability

The code used for this analysis can be found on Github at https://github.com/EPPIcenter/scale-it/ (DOI:10.5281/zenodo.4695335)^26^.
