# Supplementary material for: Citywide serosurveillance of the initial SARS-CoV-2 outbreak in San Francisco using electronic health records
Source: Nat Commun. 2021 Jun 11;12:3566. doi: 10.1038/s41467-021-23651-6 (PMC8195995; doi:10.1038/s41467-021-23651-6)
Supplement: Supplementary file 1 — Supplementary Information [file 41467_2021_23651_MOESM1_ESM.pdf]

Supplementary Information: Citywide serosurveillance of the initial SARS-CoV-2 outbreak in San Francisco using electronic health records

Isobel Routledge, Adrienne Epstein, Saki Takahashi *et al*.

Contents

Supplementary Figure 1: Histogram of samples collected by epidemiological week

Supplementary Figure 2: Plots showing, for each month of sample collection, the proportions of samples collected in each demographic category

Supplementary Figure 3: Smoothed plot showing raw seroprevalence estimates by epidemiological week.

Supplementary Table 1: Estimates of test performance characteristics from the overall procedure.

Supplementary Table 2: Seroprevalence stratified by demographic group

Supplementary Table 3: Seroprevalence stratified by neighborhood

Supplementary Table 4: Proportion of monthly sample by demographic group

Supplementary Table 5: Positive control validation samples and test results by assay.

Supplementary Table 6: Negative control validation samples and test results by assay.

Supplementary Methods 1: Estimating test performance and calulating underascertainment

Supplementary References

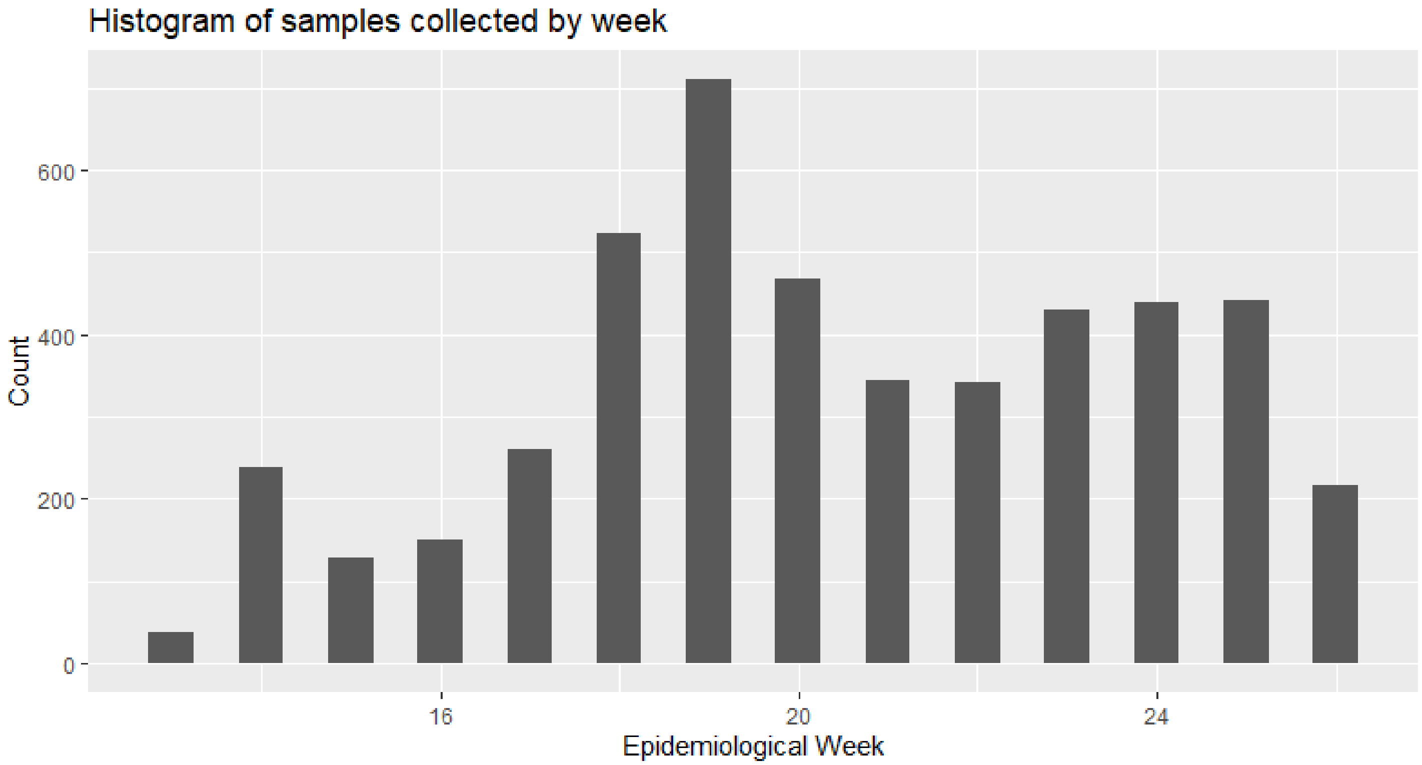

Supplementary Figure 1: Histogram of samples collected by epidemiological week. Histogram showing the count of samples collected by epidemiological week during the study,

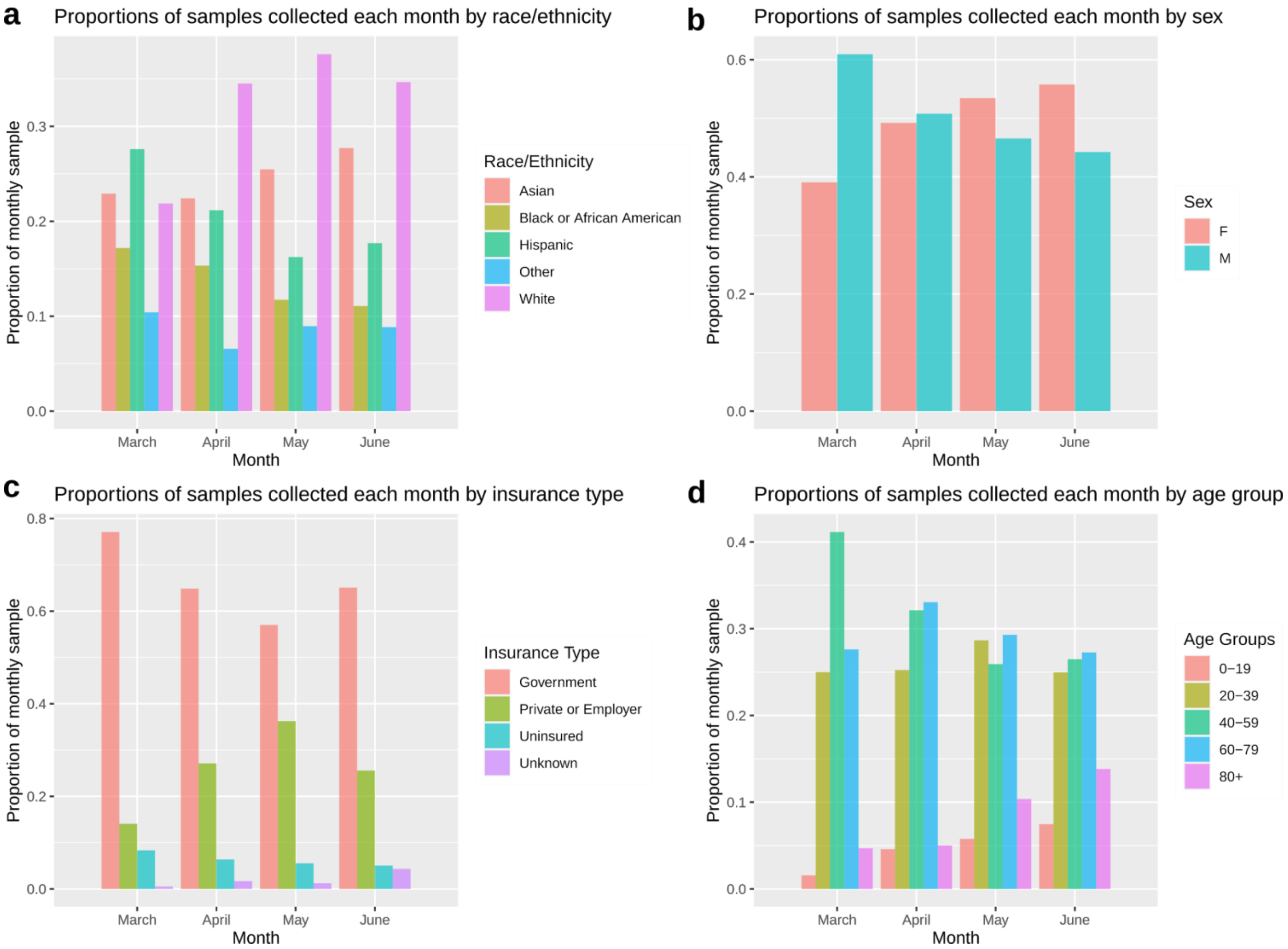

Supplementary Figure 2: Distributions of samples by demographic group and month. Plots showing, for each month of sample collection, the proportions of samples collected in each demographic category for a) Race/Ethnicity , b) Sex , c) Insurance Type and d) Age Group. Note in Figure 2b there were 13 samples with sex classified as unknown who were excluded from this analysis.

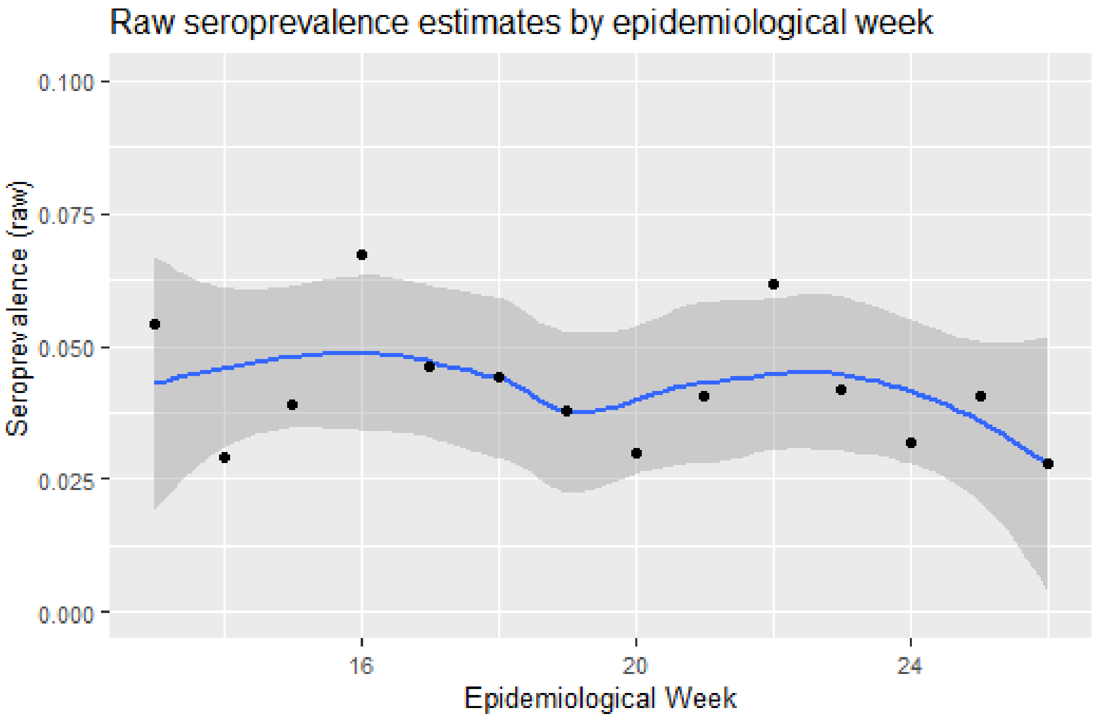

Supplementary Figure 3: Smoothed plot showing raw seroprevalence estimates by epidemiological week. Blue line shows median of smoothed loess curve and shaded grey area shows 95% confidence intervals of the fit, calculated around the mean. Points show raw seroprevalence estimates by epidemiological week.

Supplementary Table 1: Estimates of test performance characteristics from the overall procedure.

Parameter	Point estimate and 95% CrI
Se_{ELISA}	mean = 97.4%, median = 97.6%, 95% CrI = 93.9%, 99.4%
$Se_{Luminex}$	mean = 95.0%, median = 95.2%, 95% CrI = 90.9%, 98.0%
Sp_{ELISA}	mean = 97.5%, median = 97.8%, 95% CrI = 93.7%, 99.6%
$Sp_{Luminex}$	mean = 99.2%, median = 99.4%, 95% CrI = 97.0%, 100.0%
$covariance_{Se}$	mean = 1.3%, median = 1.0%, 95% CrI = -0.04%, 3.9%
$covariance_{Sp}$	mean = 0.4%, median = 0.2%, 95% CrI = 0.0%, 1.7%
$Se_{overall}$	mean = 93.7%, median = 94.0%, 95% CrI = 89.0%, 97.2%
$Sp_{overall}$	mean = 99.6%, median = 99.8%, 95% CrI = 98.2%, 100.0%
Adjusted seroprevalence (overall)	mean = 4.3%, median = 4.2%, 95% CrI = 2.1%, 6.3%

Supplementary Table 2: Seroprevalence stratified by demographic group

Table showing raw seroprevalence and point estimates and 95% credible intervals for posterior estimates of seroprevalence adjusted for test performance by demographic group.

Class	Group	Positive	Count	Seroprevalence	Adjusted seroprevalence	2.5% Credible Interval	97.5% Credible Interval
Sex	F	72	2491	0.0289	0.0270	0.0109	0.0361
Sex	M	119	2231	0.0533	0.0531	0.0373	0.0661
Age Group	0-19	11	281	0.0391	0.0410	0.0160	0.0690
Age Group	20-39	51	1261	0.0404	0.0399	0.0229	0.0545
Age Group	40-59	72	1322	0.0545	0.0546	0.0364	0.0713
Age Group	60-79	41	1390	0.0295	0.0281	0.0110	0.0404
Age Group	80+	17	481	0.0353	0.0363	0.0155	0.0580
Insurance Type	Government	120	2937	0.0409	0.0398	0.0232	0.0504
Insurance Type	Private or Employer	50	1421	0.0352	0.0341	0.0157	0.0474
Insurance Type	Uninsured	16	269	0.0595	0.0634	0.0315	0.0990
Insurance Type	Unknown	6	108	0.0556	0.0643	0.0228	0.1231
Hospital Group	UCSF	119	3037	0.0392	0.0379	0.0208	0.0486
Hospital Group	ZSFG	73	1698	0.0430	0.0419	0.0244	0.0557
Race/ Ethnicity	Asian	30	1206	0.0249	0.0233	0.0079	0.0353
Race/ Ethnicity	Black or African American	28	591	0.0474	0.0483	0.0281	0.0696
Race/ Ethnicity	Hispanic	53	858	0.0618	0.0633	0.0437	0.0829
Race/ Ethnicity	Other	14	348	0.0402	0.0422	0.0202	0.0691
Race/ Ethnicity	White	65	1675	0.0388	0.0379	0.0230	0.0506
Month	March	6	192	0.0313	0.0341	0.0097	0.0655
Month	April	44	959	0.0459	0.0459	0.0274	0.0631
Month	May	85	2047	0.0415	0.0404	0.0240	0.0529
Month	June	56	1526	0.0367	0.0358	0.0189	0.0490

Supplementary Table 3: Seroprevalence stratified by neighborhood

Table showing raw seroprevalence and point estimates and 95% credible intervals for posterior estimates of seroprevalence adjusted for test performance by neighborhood and for individuals experiencing homelessness. Adjusted seroprevalence was not estimated for neighborhoods with sample sizes below 50 samples from unique individuals.

Neighborhood	Positive	Count	Seroprevalence	Adjusted seroprevalence	2.5% Credible Interval	97.5% Credible Interval
Bayview	15	206	0.0728	0.0809	0.0456	0.1232
Bernal Heights	2	103	0.0194	0.0287	0.0040	0.0734
Castro/Upper Market	3	102	0.0294	0.0393	0.0089	0.0826
Chinatown	2	42	0.0476	NA	NA	NA
Crocker Amazon	4	49	0.0816	NA	NA	NA
Diamond Heights	1	14	0.0714	NA	NA	NA
Downtown/Civic Center	14	277	0.0505	0.0571	0.0305	0.0900
Excelsior	15	200	0.0750	0.0834	0.0450	0.1280
Financial District	1	51	0.0196	0.0387	0.0023	0.1053
Glen Park	3	37	0.0811	NA	NA	NA
Golden Gate Park	0	1	0.0000	NA	NA	NA
Haight Ashbury	2	95	0.0211	0.0310	0.0034	0.0796
Inner Richmond	4	144	0.0278	0.0340	0.0089	0.0721
Inner Sunset	8	157	0.0510	0.0580	0.0240	0.0988
Lakeshore	3	105	0.0286	0.0383	0.0095	0.0857
Marina	1	63	0.0159	0.0309	0.0019	0.0902
Mission	11	342	0.0322	0.0327	0.0139	0.0550
Nob Hill	1	80	0.0125	0.0245	0.0016	0.0685
Noe Valley	2	115	0.0174	0.0256	0.0028	0.0656
North Beach	4	80	0.0500	0.0615	0.0187	0.1282
Ocean View	5	140	0.0357	0.0431	0.0125	0.0841
Outer Mission	5	127	0.0394	0.0469	0.0160	0.0875
Outer Richmond	7	166	0.0422	0.0485	0.0192	0.0919
Outer Sunset	8	273	0.0293	0.0326	0.0121	0.0574
Pacific Heights	6	89	0.0674	0.0800	0.0309	0.1471
Parkside	5	144	0.0347	0.0413	0.0138	0.0833
Potrero Hill	5	88	0.0568	0.0699	0.0243	0.1318
Presidio	0	18	0.0000	NA	NA	NA
Presidio Heights	0	39	0.0000	NA	NA	NA
Russian Hill	0	49	0.0000	NA	NA	NA
Seacliff	0	15	0.0000	NA	NA	NA
South of Market	13	346	0.0376	0.0405	0.0197	0.0654
Twin Peaks	3	46	0.0652	NA	NA	NA
Visitacion Valley	4	121	0.0331	0.0410	0.0114	0.0835
West of Twin Peaks	5	141	0.0355	0.0427	0.0144	0.0855
Western Addition	5	305	0.0164	0.0221	0.0064	0.0434
Homeless	16	157	0.1019	0.1078	0.0614	0.1652

Supplementary Table 4: Proportion of monthly sample by demographic group. Total proportion shows proportion of total sample collected each month.

Class	Group	March	April	May	June
Sex	F	0.391	0.492	0.534	0.558
Sex	M	0.609	0.508	0.466	0.442
Age Group	0-19	0.016	0.046	0.058	0.075
Age Group	20-39	0.250	0.252	0.287	0.250
Age Group	40-59	0.411	0.321	0.259	0.265
Age Group	60-79	0.276	0.331	0.293	0.273
Age Group	80+	0.047	0.050	0.104	0.138
Insurance Type	Government	0.771	0.219	0.066	0.162
Insurance Type	Private or Employer	0.141	0.224	0.345	0.089
Insurance Type	Uninsured	0.083	0.153	0.255	0.376
Insurance Type	Unknown	0.005	0.212	0.117	0.277
Race/ Ethnicity	Asian	0.229	0.224	0.255	0.277
Race/ Ethnicity	Black or African American	0.172	0.153	0.117	0.111
Race/ Ethnicity	Hispanic	0.276	0.212	0.162	0.177
Race/ Ethnicity	Other	0.104	0.066	0.089	0.088
Race/ Ethnicity	White	0.219	0.345	0.376	0.347
Total proportion	Total proportion	0.041	0.203	0.433	0.323

Supplementary Methods 1: Estimating test performance and calulating underascertainment

Estimating test performance and positivity cutoffs for the serological assays

We employed a statistical model to estimate test performance and positive cutoffs for the two serological assays used in this study. This approach allows us to incorporate control samples that were tested on just one assay, as well as to properly account for sources of uncertainty and the covariances between the two assays.

Selecting SCALE-IT samples for confirmatory testing: All 5,244 SCALE-IT laboratory samples (corresponding to 4,735 unique patients) were first screened on the ELISA platform. In addition, 117 positive control samples from the LIINC cohort and 93 negative control samples were tested on this platform for determining assay sensitivity and specificity (Supplementary Tables 5 and 6). The antibody concentration of each sample was calculated from the ELISA OD value using a plate-specific standard curve from serial dilutions of a pool of positive control samples. Based on the distributions of concentration values among these control samples, SCALE-IT samples with an ELISA concentration value above 0.049 were selected for confirmatory testing. Based on the 117 positive and 93 negative control samples tested on this platform, this ELISA concentration cutoff corresponded to test performance characteristics of 98.3% sensitivity and 97.8% specificity.

Determining seropositivity of SCALE-IT samples: Based on the screening with the ELISA platform, 653 SCALE-IT samples were selected for confirmatory testing on the Luminex platform, on which we included three SARS-CoV-2 antigens (one preparation each of the S, RBD, and N proteins). In addition, 260 positive control samples from the LIINC cohort and 114 negative control samples were tested on this platform for determining assay sensitivity and specificity (Supplementary Tables 5 and 6). The antibody concentration of each antigen of each sample was calculated from the Luminex MFI value using a plate-specific standard curve from serial dilutions of a pool of positive control samples.

We then fit a multiple logistic regression model to the positive and negative control samples (Y=1 and Y=0, respectively) and each of their Luminex concentration values for the three antigens to determine a cutoff for positivity based on predicted probability values, to be applied to the SCALE-IT samples:

$$\log\left(\frac{p(X)}{1-p(X)}\right) = \beta_0 + \beta_S X_S + \beta_{\text{RBD}} X_{\text{RBD}} + \beta_N X_N \qquad \text{(Equation 1)}$$

The left-hand side of Equation 1 represents the logit transform of $p(X) = Pr(Y = 1|X)$, and $X = (X_s, X_{\text{RBD}}, X_N)$ are 3 predictors representing the Luminex concentration values of the S, RBD, and N antigens, respectively. The β values are estimated from the logistic regression model. Using these estimates, the predicted probability of a sample with measured concentration values X being positive can be calculated as:

$$\hat{p}(X) = \frac{\exp(\hat{\beta}_0 + \hat{\beta}_S X_S + \hat{\beta}_{\text{RBD}} X_{\text{RBD}} + \hat{\beta}_N X_N)}{1+\exp(\hat{\beta}_0 + \hat{\beta}_S X_S + \hat{\beta}_{\text{RBD}} X_{\text{RBD}} + \hat{\beta}_N X_N)} \qquad \text{(Equation 2)}$$

We determined that SCALE-IT samples with a predicted probability value of greater than or equal to 0.682 using Equation 2 would be classified as seropositive; SCALE-IT samples with a predicted probability value less than 0.682 would be classified as seronegative. This cutoff was determined as the highest predicted probability value using Luminex concentration values among the 114 negative control samples tested on this platform; this is analogous to fixing specificity at 100.0%. We chose to fix the specificity at 100.0% in order to minimize the potential for false positive results in this setting of relatively low expected seropositivity in the population. This specificity of 100.0% corresponded to a sensitivity of 95.8% (AUC: 0.983) among the 260 positive control samples tested on this platform. The five-fold cross-validated sensitivity of this algorithm, again fixing specificity at 100.0%, was estimated to be 95.4%.

Determining the test performance characteristics of a single assay: The test performance characteristics of a single assay (i.e., sensitivity (Se) and specificity (Sp)) can be determined from a 2x2 table of positive/negative control samples and their binary classification on that assay using a binomial model[1] as follows:

$$y_{\text{pos}} \sim \text{Binomial}(N_{\text{pos}}, \text{Se}) \qquad \text{(Equation 3a)}$$

$$y_{\text{neg}} \sim \text{Binomial}(N_{\text{neg}}, \text{Sp}) \qquad \text{(Equation 3b)}$$

In Equation 3a, N_{pos} represents the total number of positive control samples tested and y_{pos} represents the number of positive control samples that tested positive. In Equation 3b, N_{neg} represents the total number of negative control samples tested and y_{neg} represents the number of negative control samples that tested negative.

For a given serosurvey where N_{unknown} total samples were tested and y_{unknown} samples tested positive, the adjusted seroprevalence p_{adj} can be estimated using the binomial model in Equation 3c:

$$y_{\mathrm{unknown}} \sim \mathrm{Binomial}(N_{\mathrm{unknown}}, p_{\mathrm{adj}} \cdot \mathrm{Se} + (1 - p_{\mathrm{adj}}) \cdot (1 - \mathrm{Sp})) \qquad \text{(Equation 3c)}$$

<u>Determining the test performance characteristics of the overall serial, two-assay procedure:</u> For a two-assay scenario, the binomial model above can be extended to a multinomial framework where each control sample has two test results: their binary classification on each of the two assays. Importantly, there may be conditional dependence between assays, where conditional on the true disease status of a given sample, the test performance of one assay may vary depending on the result on the other assay. The magnitude of this conditional dependence between two assays can be directly estimated based on the results of control samples that have been tested on both assays. Here, we implemented a method developed in [2] as follows:

$$(y_{+,+|\mathrm{pos}}\ y_{+,-|\mathrm{pos}}\ y_{-,+|\mathrm{pos}}\ y_{-,-|\mathrm{pos}}) \sim \mathrm{Multinomial}((\mathrm{Se_{ELISA}} \cdot \mathrm{Se_{Luminex}}) + \mathrm{covariance_{Se}}\ (\mathrm{Se_{ELISA}} \cdot (1 - \mathrm{Se_{Luminex}}))\ - \mathrm{covariance_{Se}}\ ((1 - \mathrm{Se_{ELISA}}) \cdot \mathrm{Se_{Luminex}})\ - \mathrm{covariance_{Se}}\ ((1 - \mathrm{Se_{ELISA}}) \cdot (1 - \mathrm{Se_{Luminex}}))\ + \mathrm{covariance_{Se}}\ )$$

(Equation 4a)

$$(y_{+,+|\mathrm{neg}}\ y_{+,-|\mathrm{neg}}\ y_{-,+|\mathrm{neg}}\ y_{-,-|\mathrm{neg}}) \sim \mathrm{Multinomial}\Big(((1 - \mathrm{Sp_{ELISA}}) \cdot (1 - \mathrm{Sp_{Luminex}})) + \mathrm{covariance_{Sp}}\ ((1 - \mathrm{Sp_{ELISA}}) \cdot \mathrm{Sp_{Luminex}})\ - \mathrm{covariance_{Sp}}\ (\mathrm{Sp_{ELISA}} \cdot (1 - \mathrm{Sp_{Luminex}})) - \mathrm{covariance_{Sp}}\ (\mathrm{Sp_{ELISA}} \cdot \mathrm{Sp_{Luminex}}) + \mathrm{covariance_{Sp}}\ \Big)$$

(Equation 4b)

In Equations 4a and 4b above, the parameters refer to assay-specific sensitivities ($\mathrm{Se_{ELISA}}$ and $\mathrm{Se_{Luminex}}$), assay-specific specificities ($\mathrm{Sp_{ELISA}}$ and $\mathrm{Sp_{Luminex}}$), conditional covariance between sensitivities ($\mathrm{covariance_{Se}}$), and conditional covariance between specificities ($\mathrm{covariance_{Sp}}$). The sign of the conditional covariance value reflects the direction of the dependence in that test performance characteristic[2]. The notation $y_{(\mathrm{result\ 1}),(\mathrm{result\ 2})|(\mathrm{true\ status})}$ refers to the number of control samples with true status of positive or negative, with a result on the first assay of positive or negative (result 1) and a result on the first assay of positive or negative (result 2).

These 6 parameters were estimated by fitting Equations 4a and 4b simultaneously in a joint Bayesian model. Control samples that were tested on both assays contributed to the estimation of both assays' performance characteristics using the multinomial model (Equations 4a and 4b). We also allowed control samples that were tested only on one assay to contribute to the estimation of that assay's performance characteristics using the binomial model; in practice, this involves incorporating Equations 3a and 3b into the joint model for those control samples only. Supplementary Tables 5 and 6 below summarize the test results for our positive and negative control samples, respectively:

		Luminex (total tested on assay: 260)			
		Negative	Positive	Not Tested	Total
ELISA (total tested on assay: 117)	Negative	2	0	0	2
	Positive	1	108	6	115
	Not Tested	8	141	--	149
	Total	11	249	6	266

Supplementary Table 5: Positive control validation samples and test results by assay.

		Luminex assay			
		Negative	Positive	Not Tested	Total
ELISA assay	Negative	87	0	4	91
	Positive	1	0	1	2
	Not Tested	26	0	--	26
	Total	114	0	5	119

Supplementary Table 6: Negative control validation samples and test results by assay.

As the SCALE-IT samples were tested in a serial procedure that required a sample to be positive on the two assays to be classified as seropositive (as opposed to a parallel procedure[3]), we estimated the overall sensitivity and specificity of the approach as:

$$Se_{overall} = Se_{ELISA} \cdot Se_{Luminex} + covariance_{Se} \quad \text{(Equation 5a)}$$

$$Sp_{overall} = 1 - ((1 - Sp_{ELISA}) \cdot (1 - Sp_{Luminex})) - covariance_{Sp} \quad \text{(Equation 5b)}$$

Estimating seroprevalence, adjusting for the overall performance of the serial, two-assay procedure: Using these estimates of overall sensitivity and specificity from Equations 5a and 5b, we obtained adjusted estimates of seroprevalence (p_{adj}) as in [1], which is equivalent to Equation 3c except now replacing *Se* and *Sp* with $Se_{overall}$ and $Sp_{overall}$ derived above:

$$p_{adj} = (p_{raw} + Sp_{overall} - 1) / (Se_{overall} + Sp_{overall} - 1) \quad \text{(Equation 5c)}$$

The raw prevalence (p_{raw}) in Equation 5c refers to the proportion of SCALE-IT samples that were deemed as seropositive on the Luminex assay. The posterior estimates of these parameters are provided in Supplementary Table 1. The code to implement this model is included in our GitHub repository (https://github.com/EPPIcenter/scale-it/).

Calculating under-ascertainment

We compared our seroprevalence estimates to the weighted average of weekly cumulative incidence of reported cases up until June 14 2020 from the San Francisco Department of Public Health[4] and using the estimated population size of 881,549 in San Francisco according to the 2019 American Community Survey[5]. To estimate the proportion of overall infections that are ascertained, we weighted the weekly cumulative case counts by the proportion of our sample sampled in the corresponding week, lagged by two weeks collection to reflect the approximate time to sero-conversion among newly infected individuals[6]. We then divided the weighted average of weekly cumulative incidence of reported cases by the estimated incidence given a seroprevalence estimate of 4.2% and a population size of 881,549 .

Supplementary References

1. Rogan, W. J., & Gladen, B. Estimating prevalence from the results of a screening test. American journal of epidemiology, 107(1), 71–76 (1978)

2. Gardner, I. A., Stryhn, H., Lind, P. & Collins, M. T. Conditional dependence between tests affects the diagnosis and surveillance of animal diseases. Prev. Vet. Med. 45, 107–122 (2000)

3. de Arruda, M. M. et al. Sensitivity and specificity of parallel or serial serological testing for detection of canine Leishmania infection. Mem. Inst. Oswaldo Cruz 111, 168–173 (2016)

4. [Data] City and County of San Francisco. COVID-19 Cases and Deaths. Available at: https://data.sfgov.org/stories/s/dak2-gvuj, 2021

5. [Data] United States Census Bureau. American Community Survey Data Releases. Available at: https://www.census.gov/programs-surveys/acs/news/data-releases.html, 2021

6. Long, Q.-X. et al. Antibody responses to SARS-CoV-2 in patients with COVID-19. Nat. Med. 26, 845–848 (2020).
