# Supplementary material for: Citywide serosurveillance of the initial SARS-CoV-2 outbreak in San Francisco using electronic health records
Source: Nat Commun. 2021 Jun 11;12:3566. doi: 10.1038/s41467-021-23651-6 (PMC8195995; doi:10.1038/s41467-021-23651-6)
Supplement: Supplementary file 2 — Descriptions of Additional Supplementary Files [file 41467_2021_23651_MOESM2_ESM.pdf]

Descriptions of Additional Supplementary Files

Supplementary Data 1
Desscription: Seroprevalence stratified by demographic group. Table showing raw seroprevalence and point estimates and 95% credible intervals for posterior estimates of seroprevalence adjusted for test performance by demographic group.

Supplementary Data 2
Description: Seroprevalence stratified by neighbourhood. Table showing raw seroprevalence and point estimates and 95% credible intervals for posterior estimates of seroprevalence adjusted for test performance by neighborhood and for individuals experiencing homelessness. Adjusted seroprevalence was not estimated for neighborhoods with sample sizes below 50 samples from unique individuals.
