# Supplementary material for: Citywide serosurveillance of the initial SARS-CoV-2 outbreak in San Francisco using electronic health records
Source: Nat Commun. 2021 Jun 11;12:3566. doi: 10.1038/s41467-021-23651-6 (PMC8195995; doi:10.1038/s41467-021-23651-6)
Supplement: Supplementary file 5 — Reporting Summary [file 41467_2021_23651_MOESM5_ESM.pdf]

nature research

Corresponding author(s): Isobel Routledge

Last updated by author(s): 22-Apr-21

Reporting Summary

Nature Research wishes to improve the reproducibility of the work that we publish. This form provides structure for consistency and transparency in reporting. For further information on Nature Research policies, see our Editorial Policies and the Editorial Policy Checklist.

Statistics

For all statistical analyses, confirm that the following items are present in the figure legend, table legend, main text, or Methods section.

n/a	Confirmed	
☐	☒	The exact sample size (n) for each experimental group/condition, given as a discrete number and unit of measurement
☐	☒	A statement on whether measurements were taken from distinct samples or whether the same sample was measured repeatedly
☒	☐	The statistical test(s) used AND whether they are one- or two-sided<br>*Only common tests should be described solely by name; describe more complex techniques in the Methods section.*
☐	☒	A description of all covariates tested
☐	☒	A description of any assumptions or corrections, such as tests of normality and adjustment for multiple comparisons
☐	☒	A full description of the statistical parameters including central tendency (e.g. means) or other basic estimates (e.g. regression coefficient) AND variation (e.g. standard deviation) or associated estimates of uncertainty (e.g. confidence intervals)
☒	☐	For null hypothesis testing, the test statistic (e.g. F, t, r) with confidence intervals, effect sizes, degrees of freedom and P value noted<br>*Give P values as exact values whenever suitable.*
☒	☐	For Bayesian analysis, information on the choice of priors and Markov chain Monte Carlo settings
☒	☐	For hierarchical and complex designs, identification of the appropriate level for tests and full reporting of outcomes
☒	☐	Estimates of effect sizes (e.g. Cohen's d, Pearson's r), indicating how they were calculated

Our web collection on statistics for biologists contains articles on many of the points above.

Software and code

Policy information about availability of computer code

Data collection	Our sampling algorithm used to select samples was written in R version 4.0. It is available at https://github.com/EPPIcenter/scale-it/
Data analysis	All analysis was carried out in R version 4.0, RStan version 2.21.1 , and the google geocoding API via the ggmap R package. The visualization of the maps in Figure 3 were produced in QGIS version 3.12.2. Code used to produce posterior seroprevalence estimates (stratified and overall) is available at https://github.com/EPPIcenter/scale-it/

For manuscripts utilizing custom algorithms or software that are central to the research but not yet described in published literature, software must be made available to editors and reviewers. We strongly encourage code deposition in a community repository (e.g. GitHub). See the Nature Research guidelines for submitting code & software for further information.

Data

Policy information about availability of data

All manuscripts must include a data availability statement. This statement should provide the following information, where applicable:

- Accession codes, unique identifiers, or web links for publicly available datasets
- A list of figures that have associated raw data
- A description of any restrictions on data availability

To avoid identifiability of data and to comply with institutional policy around data privacy, we have provided summarized data by demographic group and neighborhood instead of individual-level data, used to generate figure 4a-b, as well as posterior values for seroprevalence by demographic group used to generate figure 3a-d. The aggregated data used for this analysis can be found on Github at https://github.com/EPPIcenter/scale-it/ (DOI:10.5281/zenodo.4695335)28. Maps were created in QGIS (QGIS.org, QGIS Geographic Information System. QGIS Association. http://www.qgis.org , 2021) using shapefiles in the public domain (Figure 2c: California. Metropolitan Transportation Commission. Census Zip Code Tabulation Areas, 2000 - San Francisco Bay Area, California. Retrieved from https://earthworks.stanford.edu/catalog/stanford-df986nv4623, 2002) (Figure 4a-d: City of San Francisco, SF data (2019) Planning Neighborhood Groups Map, https://

data.sfgov.org/Geographic-Locations-and-Boundaries/Planning-Neighborhood-Groups-Map/iacs-ws63 , 2019). Cumulative incidence by planning neighborhood from March - June 2020 in Figure 4c used publicly available data from the San Francisco department of Public Health (https://data.sfgov.org/COVID-19/COVID-19-Cases-by-Geography-and-Date/d2ef-idww). Figures 3 and 4 visualize Supplementary Tables 2 and 3. Figure 2 visualizes the distribution of samples, although because the underlying raw data for Figure 2 are at the individual level, they have not been shared with the manuscript for ethical reasons, although the summarized demographic distributions of the samples are included in the manuscript (Table 1).

Field-specific reporting

Please select the one below that is the best fit for your research. If you are not sure, read the appropriate sections before making your selection.

☒ Life sciences ☐ Behavioural & social sciences ☐ Ecological, evolutionary & environmental sciences

For a reference copy of the document with all sections, see nature.com/documents/nr-reporting-summary-flat.pdf

Life sciences study design

All studies must disclose on these points even when the disclosure is negative.

Sample size	We collected an average of 1510 samples per month. We determined this sample size based on considerations of both statistical power and feasibility. To estimate seroprevalence with an absolute error of S% and at Type I error of 5%, and a prior of 20% seroprevalence, a sample size of 246 individuals would need to be tested each month. We determined that an overall sample size of a minimum 1230 samples per month would be sufficient to allow stratification of results by five age groups (0-19, 20-39, 40-59, 60-79, 80+ years).
Data exclusions	From the full list of residual serum samples that were available, we restricted our sampling frame to samples from individuals undergoing routine blood testing, to target a population which was more representative of community transmission, rather than oversampling from a population which may be seriously ill. We included patients residing in San Francisco, including those experiencing homelessness. We excluded individuals who were tested for SARS-CoV-2 during the visit when they received their blood draw (except if the test was for routine purposes, such as testing prior to an elective procedure or admittance to the hospital). This was to reduce bias in our sample related to treatment seeking behaviour. We restricted our sample to outpatient and emergency department visits for adults; to have a sample which was more representative of community transmission. For the youngest age group, we included both inpatient and outpatient visits due to small numbers of available samples. Finally, we excluded samples if samples from the same patient had been selected within the previous 30 days, to avoid oversampling the same individual.<br><br>After obtaining the list of eligible samples according to the above criteria, we selected serum samples for the study using a sampling algorithm aimed to ensure an adequate sample size for each of five age strata and to maximize geographic representativity. After setting a daily target sample size for our overall population, we divided this equally between five age bins to set a target sample size for each age bin. We also set a target sample size for each zip code which was proportional to its population size. For each zipcode with a larger number of eligible samples than its target size, we kept all samples from age groups with sample sizes below or at their target and obtained a random sample from any age group that had an eligible sample size above the target size. We intentionally over-sampled pregnant women as a healthy sentinel population by aiming to obtain 10% of the samples from pregnant women undergoing routine care.<br><br>When analysing only samples from unique individuals, where there were two samples originating from the same person, one was excluded at random. All of the stated exclusion criteria were pre-established at the study design stage.
Replication	All samples were screened using an in-house ELISA assay which was performed once per sample, and then confirmatory testing was performed once on a subset of samples above a threshold value using an in-house multiplex microsphere assay (Luminex platform) to detect IgG against the SARS-CoV-2 S protein, RBD, and the nucleocapsid (N) protein. A logistic regression model including the concentration values of the three antigens for each sample was determined to have the highest cross-validation accuracy for classification, and was used to establish a cutoff for positivity. We required a sample to be positive on both of the two assays to be classified as seropositive. Code used for the analysis has been made available on GitHub and produces reproducible results.
Randomization	Our study was observational so there was no assignment to groups or treatments. When samples were selected following our inclusion/exclusion criteria, samples were selected at random within their stratified groups. Our stratified sampling approach, inclusion/exclusion criteria, adjustment of estimates for assay sensitivity and specificity and weighting overall seroprevalence estimates by age and sex, all assist in controlling for covariates and reducing biases.
Blinding	There was no allocation of groups, as this was an observational study. However samples were only referred to by tube barcode and a unique patient identifier, and re-linked to demographic information once seropositivity/negativity was determined, meaning during both data collection and analysis, researchers were blinded to the demographic information associated with the samples.

Reporting for specific materials, systems and methods

We require information from authors about some types of materials, experimental systems and methods used in many studies. Here, indicate whether each material, system or method listed is relevant to your study. If you are not sure if a list item applies to your research, read the appropriate section before selecting a response.

Materials & experimental systems

n/a	Involved in the study	
[ ]	[x]	Antibodies
[x]	[ ]	Eukaryotic cell lines
[x]	[ ]	Palaeontology and archaeology
[x]	[ ]	Animals and other organisms
[ ]	[x]	Human research participants
[x]	[ ]	Clinical data
[x]	[ ]	Dual use research of concern

Methods

n/a	Involved in the study	
[x]	[ ]	ChIP-seq
[x]	[ ]	Flow cytometry
[x]	[ ]	MRI-based neuroimaging

Antibodies

Antibodies used	For the luminex assay the following secondary antibody was used: R-Phycoerythrin†-conjugated AffiniPure F(ab')2 Fragment Goat Anti-Human IgG, Fcg Fragment Specific (minimal cross-reaction to Bovine, Horse, and Mouse Serum Proteins) (catalog number 109-116-098). The dilution used was 1:400.
Validation	N/A, only secondary antibodies were used.

Human research participants

Policy information about studies involving human research participants

Population characteristics	The population characteristics are detailed in Table 1 and Figure 2 of the manuscript. Briefly, our sample population consisted of San Francisco residents, selected to stratify by age and zipcode. By sex the population was N = 2,491 (52.6%) female, N = 2,231 (47.1%) male and N=13 (0.3%) unknown sex. By age, N= 281 (5.9%) was aged 0-19, N = 1,261 (26.6%) was aged 20-39, N = 1,322 (27.9%) was aged 40-59, N = 1,390 (29.4%) was aged 60 - 79, and N= 481 (10.2%) was aged 80+.
Recruitment	Samples were obtained as residual sera taken from individuals undergoing routine blood testing, either through the UCSF Health network, or through the San Francisco Department of Public Health/Zuckerberg San Francisco General Hospital network. Therefore our study only captured those served by these hospital networks. However, the networks serve different patient populations because the ZSFGH serves underinsured or uninsured patients.
Ethics oversight	UCSF IRB #20-30379 (Serological Surveillance of SARS-CoV-2 in Residual Serum/Plasma Samples). The IRB did not require patient contact or written consent to use residual sera. The LIINC study (providing positive control samples) was approved by the UCSF (IRB #20-30479).

Note that full information on the approval of the study protocol must also be provided in the manuscript.
